# Candidate gene polymorphisms study between human African trypanosomiasis clinical phenotypes in Guinea

**DOI:** 10.1371/journal.pntd.0005833

**Published:** 2017-08-21

**Authors:** Justin Windingoudi Kaboré, Hamidou Ilboudo, Harry Noyes, Oumou Camara, Jacques Kaboré, Mamadou Camara, Mathurin Koffi, Veerle Lejon, Vincent Jamonneau, Annette MacLeod, Christiane Hertz-Fowler, Adrien Marie Gaston Belem, Enock Matovu, Bruno Bucheton, Issa Sidibe, for the TrypanoGEN Research Group as members of The H3Africa Consortium

**Affiliations:** 1 Centre International de Recherche-Développement sur l’Elevage en zone Subhumide (CIRDES), Bobo-Dioulasso, Burkina Faso; 2 Centre for Genomic Research, University of Liverpool, Liverpool, United Kingdom; 3 Programme National de Lutte contre la Trypanosomose Humaine Africaine, Conakry, Guinea; 4 Université Nazi Boni (UNB), Bobo-Dioulasso, Burkina Faso; 5 Université Jean Lorougnon Guédé (UJLoG), Daloa, Côte d’Ivoire; 6 Institut de Recherche pour le Développement (IRD), Montpellier, France; 7 Institut Pierre Richet, Bouaké, Côte d’Ivoire; 8 Wellcome Trust Centre for Molecular Parasitology, University Place, Glasgow, United Kingdom; 9 College of Veterinary Medicine, Animal Resources and Bio-security, Makerere University, Kampala, Uganda; Institut de recherche pour le developpement, FRANCE

## Abstract

**Background:**

Human African trypanosomiasis (HAT), a lethal disease induced by *Trypanosoma brucei gambiense*, has a range of clinical outcomes in its human host in West Africa: an acute form progressing rapidly to second stage, spontaneous self-cure and individuals able to regulate parasitaemia at very low levels, have all been reported from endemic foci. In order to test if this clinical diversity is influenced by host genetic determinants, the association between candidate gene polymorphisms and HAT outcome was investigated in populations from HAT active foci in Guinea.

**Methodology and results:**

Samples were collected from 425 individuals; comprising of 232 HAT cases, 79 subjects with long lasting positive and specific serology but negative parasitology and 114 endemic controls. Genotypes of 28 SNPs in eight genes passed quality control and were used for an association analysis. *IL6* rs1818879 allele *A* (p = 0.0001, OR = 0.39, CI_95_ = [0.24–0.63], BONF = 0.0034) was associated with a lower risk of progressing from latent infection to active disease. *MIF* rs36086171 allele *G* seemed to be associated with an increased risk (p = 0.0239, OR = 1.65, CI_95_ = [1.07–2.53], BONF = 0.6697) but did not remain significant after Bonferroni correction. Similarly *MIF* rs12483859 *C* allele seems be associated with latent infections (p = 0.0077, OR = 1.86, CI_95_ = [1.18–2.95], BONF = 0.2157). We confirmed earlier observations that *APOL1* G2 allele (DEL) (p = 0.0011, OR = 2.70, CI_95_ = [1.49–4.91], BONF = 0.0301) is associated with a higher risk and *APOL1* G1 polymorphism (p = 0.0005, OR = 0.45, CI_95_ = [0.29–0.70], BONF = 0.0129) with a lower risk of developing HAT. No associations were found with other candidate genes.

**Conclusion:**

Our data show that host genes are involved in modulating *Trypanosoma brucei gambiense* infection outcome in infected individuals from Guinea with *IL6* rs1818879 being associated with a lower risk of progressing to active HAT. These results enhance our understanding of host-parasite interactions and, ultimately, may lead to the development of new control tools.

## Author summary

Human African Trypanosomiasis (HAT) known as sleeping sickness is a lethal neglected disease in West and Central Africa, caused by the parasite *Trypanosoma brucei gambiense* that is transmitted by tsetse flies *Glossina palpalis gambiensis*. The disease has long been considered to be invariably fatal, but field studies show that *T*. *b*. *gambiense* infection leads to a wide diversity of clinical outcomes. An acute form progressing rapidly to second stage; spontaneous self-cure and individuals able to control parasitaemia at very low levels (latent infections). In the present study, we test for associations between candidate gene polymorphisms and different HAT phenotypes (HAT confirmed cases, latent infections) and endemic controls, in order to identify relationships between the clinical diversity and host genetic determinants. Our results, based on clinical, serological and parasitological observations, combined with genotypes and association analysis form the most complete study of host genetic determinants and clinical diversity of HAT in Guinea to date. We show that host genes are involved in modulating responses in *Trypanosoma brucei gambiense* infected individuals. A new association was found between *IL6* rs1818879 and a reduced risk of progressing from latent infection to active HAT. These results constitute an important stage toward the identification of the natural human mechanisms involved in *T*. *b*. *gambiense* infection regulation.

## Introduction

Human African trypanosomiasis (HAT) known as sleeping sickness is a neglected disease of sub-Saharan Africa caused by two sub-species of trypanosomes, *Trypanosoma brucei* (*T*. *b*.) *gambiense* (in West and Central Africa) and *T*. *b*. *rhodesiense* (in East and South Africa), with *T*. *b*. *gambiense* causing > 95% of all cases [1]. In West Africa, Guinea is the country with the highest prevalence for HAT, especially on the coast [2], where the vector, the tsetse fly *Glossina palpalis gambiensis* is abundant [3]. In the active foci of Boffa, Dubreka and Forecariah prevalence in humans is generally around 0.5–1%, but can go up to 5% in some villages [1,3,4]. HAT caused by *T*. *b*. *gambiense* is classically described as a chronic disease with an early haemolymphatic phase (first stage) associated with nonspecific symptoms such as intermittent fevers and headaches, followed by a meningoencephalitic phase (second stage) where the parasite invades the central nervous system (CNS) leading to neurological disorders. In the absence of treatment, HAT is widely assumed to be 100% fatal. However, asymptomatic carriers and spontaneous cure without treatment have been described in old [5] and more recent reports [6], strengthening the evidence for human trypanotolerance / resistance [7–10]. Indeed, a recent long-term longitudinal survey in Côte d’Ivoire found people who were initially diagnosed by microscopy but on follow-up examination, up to 15 years later, had no detectable parasitaemia by microscopy, despite not having received treatment [6]. A drop in antibody titers to seronegative levels was detected in some of these subjects, indicating that they have self-cured. In contrast, others maintained a long-lasting serological response, being Card Agglutination Test for Trypanosomiasis (CATT) and trypanolysis (TL) test positive but had no parasites detectable by microscopy, suggesting that these individuals were able to control blood parasitaemia at very low levels and were considered as asymptomatic carriers of parasites and were classified as latent infections [4,9,11].

Many factors could play a role in this variability of response to infection, and the respective roles of the virulence of the parasite and host susceptibility in this clinical diversity remain unclear [12]. It has been suggested that genetic polymorphism of the parasite could be associated with asymptomatic and very chronic infections [11]. Nevertheless, host genetic factors involved in the control of immunity could regulate infection levels or mortality rates, as has been shown for *Trypanosoma congolense* infections in experimental models [13,14] and also *T*. *brucei spp* in humans [15–20].

Hence, the purpose of the present study was to study the role of single nucleotide polymorphisms (SNPs) in *IL4*, *IL6*, *IL8*, *IL10*, *IFNG*, *APOL1*, *TNFA*, *HPR*, *HLA-G*, *HLA-A*, *HP*, and *MIF* genes on susceptibility/resistance to HAT by means of an association study between HAT cases, seropositive microscopically aparasitaemic subjects with latent infections, and controls in order to explore their possible role in human immunity to this complex disease.

## Methods

### Informed consent and ethics statement

The study was performed as part of medical survey conducted by the national control program according to the national HAT diagnostic procedures and was approved by the Ministry of Health in Guinea. All participants were informed about the objective of the study in their own language and signed an informed consent form. For participants under 18 year of age, a written informed consent was obtained from the parent. This study is part of a TrypanoGEN project which aims to understand the genetic basis of human susceptibility to trypanosomiasis and samples were archived in the TrypanoGEN Biobank at CIRDES [21] for which approval was obtained from the Guinea National ethics committee (1-22/04/2013).

### Study population

The study was carried out in three active HAT foci (Dubreka, Boffa, and Forecariah) in the mangrove areas of coastal Guinea. Most of the population is from the Soussou ethnic group and lives in small villages scattered along mangrove channels [1,3]. All subjects included in this study were identified during medical monitoring surveys organized by the National HAT Control Program (NCP) between November 2007 and December 2013, according to the WHO and NCP policies described elsewhere [4]. Blood (5 ml) was collected in heparinized tubes. For individuals who are positive to the CATT (Card Agglutination Test for Trypanosomiasis) serological mass screening test, a twofold plasma dilution series was tested to determine their CATT end titer. All individuals with titers of 1/4 or greater were submitted to microscopic examination of lymph node aspirates whenever swollen lymph nodes were present; 350 ml of buffy coat was then examined by using the mini-anion exchange column (mAECT) test which has shown to have a threshold of detection of 10 trypanosomes ml^-1^ of blood [3,22]. Samples that were CATT negative, CATT positive with lymph node and/or buffy coat negative for trypanosomes were all subject to the immune trypanolysis test (TL), which is a serological test that is highly specific for *T*. *b*. *gambiense* [23]. 425 individuals were selected according to the study inclusion criteria below.

### Phenotype definitions

Samples were classified into three phenotypes: (1) Cases or active HAT patients are defined as subjects presenting as positive on both serological tests (CATT and TL) and parasitological tests (mAECT and/or examination of cervical lymph juice aspirates); (2) latent infections have CATT plasma dilution end titer 1/4 or higher; TL positive and are parasitology negative and maintain this phenotype for at least two years; (3) endemic controls who have serology (CATT and TL) negative and living in the same village as a HAT patient and/or a seropositive subject. All individuals live in the same area and had been exposed to the risk of infection since birth [21].

### Study design

This study was one of six studies of populations of HAT endemic areas in DRC, Cameroon, Cote d’Ivoire, Guinea, Malawi and Uganda. The studies were designed to have 80% power to detect odds ratios (OR) >2 for loci with disease allele frequencies of 0.15–0.65 with the 80 SNPs genotyped. The study design included a total of 425 samples: 232 HAT cases, 79 seropositive and 114 uninfected or endemic controls. Power calculations were undertaken using the genetics analysis package gap in r [24].

### DNA extraction

DNA was extracted from buffy coat (BC) samples using the Qiagen DNA extraction kit (QIAamp DNA Blood Midi Kit) following the instructions of the manufacturer. The DNA extract was stored at -20°C. After extraction each DNA sample was quantified on a spectrophotometer (NanoDrop).

### Single Nucleotide Polymorphisms (SNPs) selection

80 SNP were selected for genotyping using two strategies: 1) specific SNP in *IL10*, *TNFA*, *HLA-A*, *HLA-G*, *APOL1*, *MIF*, *HPR* and *HP* had been previously reported to be associated with HAT or 2) *IL4*, *IL8*, *IL6*, *HLA-G* and *IFNG* were scanned for sets of linked marker SNP (r^2^ < 0.5) across each gene. The SNPs in this second group of genes were selected using a merged set of SNP obtained from low fold coverage (8-10x) whole genome shotgun data generated from 230 residents living in regions (Democratic Republic of Congo, Guinea Conakry, Ivory Coast and Uganda) where trypanosomiasis is endemic (TrypanoGEN consortium, European Nucleotide Archive Study Number EGAS00001002482) and 1000 Genomes Project data from African populations, only published SNP with dbSNP identifiers were used in the design. Linkage (r^2^) between loci was estimated using Plink and sets of SNP that covered the gene were identified. Some SNP loci were excluded during assay development or failed to genotype and were not replaced.

### Genotyping

Samples were submitted to Plateforme Genome Transcriptome de Bordeaux at INRA Site de Pierroton. Multiplex design (two sets of 40 SNPs) was performed using Assay Design Suite v2.0 (Agena Biosciences). SNP genotyping was achieved with the iPLEX Gold genotyping kit (Agena Biosciences) for the MassArray iPLEX genotyping assay, following the manufacturer’s instructions. Products were detected on a MassArray mass spectrophotometer and data were acquired in real time with MassArray RT software (Agena Biosciences). SNP clustering and validation was carried out with Typer 4.0 software (Agena Biosciences). *APOL1* rs71785313 SNP was genotyped again by LGC Genomics, Hoddesden, United Kingdom, using the PCR based KASP assay [25].

### Statistical analysis

Plink v1.9 [26] was used for statistical analysis, allele frequencies were analyzed by simple allele counting and the R 3.3.1 software package was used for data visualization (R Foundation for Statistical Computing, Vienna Austria). For quality control and filtering, SNPs loci with missing genotypes > 10% and individuals with missing loci > 10% were removed. In addition SNPs with Hardy equilibrium (HWE) p < 0.001, minor allele frequency MAF < 0.05, SNPs in linkage with adjacent SNPs (r^2^ > 0.5) and monomorphic loci were also pruned [27]. 28 SNPs were remaining after filtering and LD pruning and were used to test association with the disease. Association analysis’s were done using pairwise comparison between cases-controls, cases-latent infections and latent infections-controls. The Fisher exact test was used to test for significant differences in allele frequencies between phenotypes. We also tested for association with disease under additive model allowing for non-genetic risk factors “sex and age”. Odds ratio for the minor allele A1, and p-value for association, were adjusted for age and sex. In all analysis, results were adjusted by Bonferroni correction for multiple comparisons. The Bonferroni correction establishes the threshold of significance at α/n. P-values smaller than 0.05/28 = 0.0018 or an adjusted p-value <0.05 were considered significant.

## Results

### Genes and SNPs selected

In total 12 candidate genes that have known or plausible associations with HAT were identified from the literature. 80 SNPs were identified 17 in *HLA-G*, 2 in *HLA-A*, 2 in *HPR*, 10 in *IFNG*, 16 in *IL4*, 12 in *IL6*, 6 in *IL8*, 1 in *IL10*, 8 in *MIF*, 3 in *TNFA*, 1 in *HP* and 2 in *APOL1*. 28 of these 80 SNPs remained after quality control and linkage pruning and were used for association analysis (Table 1). These SNPs are in HWE, MAF > 5% and LD r^2^ < 0.5. SNPs with allele frequencies that deviated from HWE were removed. The allelic and minor allele carrier frequencies are shown in Tables 2–4, along with the results of the association test.

**Table 1:** SNPs remaining after quality control and LD pruning.

CHR	Genes	SNPs selected	SNPs pass filtered
1	*IL10*	1	0
4	*IL8*	6	4
5	*IL4*	16	3
6	*HLAG*	17	2
6	*TNFA*	3	2
6	*HLAA*	2	0
7	*IL6*	12	6
12	*IFNG*	10	5
16	*HPR*	2	0
16	*HP*	1	1
22	*MIF*	8	3
22	*APOL1*	2	2
Total	12	80	28

CHR: Chromosome number, SNP: single nucleotide polymorphism

**Table 2:** Association analysis between HAT cases and controls.

CHR	Genes	SNP	BP	A1	A2	P	BONF	FDR_BH	FRD_BY	OR	CI_95_	HWE	F_ST_
4	*IL8*	rs114259658	74605639	*A*	*T*	0.7973	1	0.9408	1	1.08	0.59–2.00	0.4410	-0.00361
4	*IL8*	rs2227307	74606669	*T*	*G*	0.2343	1	0.5468	1	0.81	0.57–1.15	0.1314	0.00104
4	*IL8*	rs2227545	74608727	*C*	*A*	0.1212	1	0.4848	1	1.63	0.88–3.01	1	0.00288
4	*IL8*	rs58478511	74610033	*A*	*G*	0.4888	1	0.8235	1	1.14	0.78–1.66	0.8098	-0.00218
5	*IL4*	rs2243261	132012806	*T*	*G*	0.1736	1	0.4860	1	0.75	0.49–1.14	0.4260	0.00326
5	*IL4*	rs2243283	132016593	*G*	*C*	0.5212	1	0.8235	1	0.87	0.56–1.34	0.2041	-0.00405
5	*IL4*	rs9282745	132014000	*A*	*T*	0.9918	1	0.9918	1	1.00	0.57–1.74	0.0706	-0.00265
6	*HLAG*	rs1610696	29798803	*G*	*C*	0.9145	1	0.9849	1	0.98	0.67–1.44	0.0016	-0.00398
6	*HLAG*	rs2517898	29799746	*G*	*C*	0.6884	1	0.9178	1	0.93	0.64–1.34	0.1739	-0.00054
6	*TNFA*	rs1800629	31543031	*A*	*G*	0.8156	1	0.9408	1	1.06	0.66–1.71	0.2207	-0.00108
6	*TNFA*	rs1800630	31542476	*A*	*C*	0.2117	1	0.5388	1	1.47	0.80–2.67	1	-0.00044
7	*IL6*	rs1474347	22768124	*C*	*A*	0.0941	1	0.4393	1	1.91	0.90–4.08	1	0.00671
7	*IL6*	rs1548216	22769773	*C*	*G*	0.2988	1	0.6025	1	1.28	0.81–2.02	1	0.00392
7	*IL6*	rs1818879	22772727	*A*	*G*	0.1698	1	0.4860	1	0.71	0.43–1.16	0.4504	0.00487
7	*IL6*	rs2066992	22768249	*T*	*G*	0.5336	1	0.8235	1	0.82	0.45–1.52	1	-0.00152
7	*IL6*	rs2069837	22768027	*G*	*A*	0.0871	1	0.4393	1	0.64	0.38–1.07	0.1147	0.00977
7	*IL6*	rs2069855	22772624	*C*	*T*	0.3012	1	0.6025	1	1.41	0.74–2.70	1	-0.00041
12	*IFNG*	rs2069705	68555011	*A*	*G*	0.9559	1	0.9913	1	1.01	0.72–1.41	0.3431	-0.00206
12	*IFNG*	rs2069722	68548953	*A*	*G*	0.5588	1	0.8235	1	1.25	0.59–2.62	1	-0.00184
12	*IFNG*	rs2069728	68547784	*T*	*C*	0.8400	1	0.9408	1	1.04	0.71–1.51	0.3854	-0.00327
12	*IFNG*	rs2430561	68552522	*A*	*T*	0.1464	1	0.4860	1	1.56	0.86–2.86	0.5307	0.00438
12	*IFNG*	rs78554979	68554636	*C*	*T*	0.0449*	1	0.4393	1	0.55	0.31–0.99	0.2102	0.00940
16	*HP*	rs8062041	72088964	*T*	*C*	0.6853	1	0.9178	1	1.07	0.76–1.51	0.6999	-0.00300
22	*APOL1*	rs71785313	36661916	DEL	INSERT	0.5324	1	0.8235	1	1.15	0.74–1.79	0.3534	-0.00147
22	*APOL1*	rs73885319	36661906	*G*	*A*	0.7318	1	0.9314	1	0.92	0.59–1.45	1	-0.00325
22	*MIF*	rs12483859	24234807	*C*	*T*	0.0807	1	0.4393	1	1.38	0.96–1.98	0.1429	0.00610
22	*MIF*	rs34383331	24238079	*A*	*T*	0.0914	1	0.4393	1	0.67	0.42–1.07	1	0.00581
22	*MIF*	rs36086171	24235455	*G*	*A*	0.0239*	0.6697	0.4393	1	1.65	1.07–2.53	0.3772	0.02344

CHR: Chromosome number, SNP: single nucleotide polymorphism, BP: Physical position (base-pair in GRCh37), A1: Minor allele name, A2: Major allele name, P: Exact p-value, BONF: Bonferroni corrected p-value, FDR: false discovery rate, OR: Estimated odds ratio (for A1), CI_95_: 95% confidence interval, HWE: Hardy-Weinberg Equilibrium p-value

* P-value significant; DEL: deletion of 6 base pair; INSER: insertion of 6 base pair.

**Table 3:** Association analysis between HAT cases and latent infection groups.

CHR	Genes	SNP	BP	A1	A2	P	BONF	FDR_BH	FRD_BY	OR	CI_95_	HWE	F_ST_
4	*IL8*	rs114259658	74605639	*A*	*T*	0.7133	1	0.8628	1	0.89	0.47–1.68	0.4410	-0.00384
4	*IL8*	rs2227307	74606669	*T*	*G*	0.4650	1	0.7931	1	1.15	0.79–1.66	0.1314	-0.00118
4	*IL8*	rs2227545	74608727	*C*	*A*	0.5518	1	0.7931	1	1.20	0.65–2.22	1	-0.00332
4	*IL8*	rs58478511	74610033	*A*	*G*	0.4429	1	0.7931	1	0.86	0.59–1.26	0.8098	0.00058
5	*IL4*	rs2243261	132012806	*T*	*G*	0.9303	1	0.9306	1	0.98	0.62–1.55	0.4260	-0.00450
5	*IL4*	rs2243283	132016593	*G*	*C*	0.5761	1	0.7931	1	0.88	0.55–1.39	0.2041	-0.00504
5	*IL4*	rs9282745	132014000	*A*	*T*	0.5372	1	0.7931	1	1.24	0.63–2.46	0.0706	-0.00376
6	*HLAG*	rs1610696	29798803	*G*	*C*	0.7436	1	0.8628	1	1.08	0.69–1.69	0.0016	-0.00462
6	*HLAG*	rs2517898	29799746	*G*	*C*	0.5946	1	0.7931	1	1.12	0.75–1.66	0.1739	-0.00272
6	*TNFA*	rs1800629	31543031	*A*	*G*	0.9306	1	0.9306	1	1.02	0.61–1.72	0.2207	-0.00388
6	*TNFA*	rs1800630	31542476	*A*	*C*	0.5948	1	0.7931	1	1.19	0.63–2.23	1	-0.00240
7	*IL6*	rs1474347	22768124	*C*	*A*	0.7960	1	0.8628	1	1.10	0.54–2.21	1	-0.00380
7	*IL6*	rs1548216	22769773	*C*	*G*	0.5169	1	0.7931	1	1.17	0.72–1.90	1	-0.00117
7	*IL6*	rs1818879	22772727	*A*	*G*	0.0001*	0.0034**	0.004	0.0133	0.39	0.24–0.63	0.4504	0.08256
7	*IL6*	rs2066992	22768249	*T*	*G*	0.1921	1	0.6455	1	0.66	0.36–1.23	1	0.00583
7	*IL6*	rs2069837	22768027	*G*	*A*	0.8012	1	0.8628	1	1.08	0.60–1.93	0.1147	-0.00391
7	*IL6*	rs2069855	22772624	*C*	*T*	0.3414	1	0.7931	1	1.41	0.70–2.85	1	-0.00194
12	*IFNG*	rs2069705	68555011	*A*	*G*	0.4609	1	0.7931	1	0.87	0.61–1.25	0.3431	-0.00382
12	*IFNG*	rs2069722	68548953	*A*	*G*	0.2072	1	0.6455	1	1.80	0.72–4.50	1	0.00331
12	*IFNG*	rs2069728	68547784	*T*	*C*	0.7288	1	0.8628	1	1.07	0.72–1.60	0.3854	-0.00384
12	*IFNG*	rs2430561	68552522	*A*	*T*	0.4592	1	0.7931	1	0.81	0.47–1.41	0.5307	-0.00261
12	*IFNG*	rs78554979	68554636	*C*	*T*	0.0762	1	0.4264	1	0.57	0.31–1.06	0.2102	0.00916
16	*HP*	rs8062041	72088964	*T*	*C*	0.3551	1	0.7931	1	1.19	0.82–1.73	0.6999	-0.00340
22	*APOL1*	rs71785313	36661916	DEL	INSERT	0.0011*	0.0301**	0.0100	0.0394	2.70	1.49–4.91	0.3534	0.04098
22	*APOL1*	rs73885319	36661906	*G*	*A*	0.0005*	0.0129**	0.0065	0.0254	0.45	0.29–0.70	1	0.05137
22	*MIF*	rs12483859	24234807	*C*	*T*	0.1643	1	0.6455	1	0.77	0.54–1.11	0.1429	0.00376
22	*MIF*	rs34383331	24238079	*A*	*T*	0.0673	1	0.4264	1	0.64	0.40–1.03	1	0.00985
22	*MIF*	rs36086171	24235455	*G*	*A*	0.2075	1	0.6455	1	1.32	0.86–2.02	0.3772	0.00459

CHR: Chromosome number, SNP: single nucleotide polymorphism, BP: Physical position (base-pair in GRCh37), A1: Minor allele name, A2: Major allele name, P: Exact p-value, BONF: Bonferroni corrected p-value, FDR: false discovery rate, OR: Estimated odds ratio (for A1), CI95: 95% confidence interval, HWE: Hardy-Weinberg Equilibrium p-value

* P-value significant

** Bonferroni correction significant

DEL: deletion of 6 base pair

INSER: insertion of 6 base pair.

**Table 4:** Association analysis between latent infection and controls groups.

CHR	Genes	SNP	BP	A1	A2	P	BONF	FDR_BH	FRD_BY	OR	CI_95_	HWE	F_ST_
4	*IL8*	rs114259658	74605639	*A*	*T*	0.6168	1	0.9090	1	1.22	0.57–2.62	0.4410	-0.00351
4	*IL8*	rs2227307	74606669	*T*	*G*	0.0728	1	0.3402	1	0.67	0.43–1.04	0.1314	0.01008
4	*IL8*	rs2227545	74608727	*C*	*A*	0.4602	1	0.8088	1	1.31	0.64–2.69	1	-0.00240
4	*IL8*	rs58478511	74610033	*A*	*G*	0.1492	1	0.5455	1	1.39	0.89–2.17	0.8098	0.00690
5	*IL4*	rs2243261	132012806	*T*	*G*	0.2078	1	0.5818	1	0.71	0.42–1.21	0.4260	-0.00060
5	*IL4*	rs2243283	132016593	*G*	*C*	0.9907	1	0.9907	1	1.00	0.59–1.70	0.2041	-0.00552
5	*IL4*	rs9282745	132014000	*A*	*T*	0.4863	1	0.8088	1	0.77	0.38–1.59	0.0706	-0.00246
6	*HLAG*	rs1610696	29798803	*G*	*C*	0.6677	1	0.9337	1	0.90	0.56–1.45	0.0016	-0.00503
6	*HLAG*	rs2517898	29799746	*G*	*C*	0.2517	1	0.6407	1	0.76	0.48–1.21	0.1739	0.00193
6	*TNFA*	rs1800629	31543031	*A*	*G*	0.8195	1	0.9337	1	1.07	0.61–1.87	0.2207	-0.00452
6	*TNFA*	rs1800630	31542476	*A*	*C*	0.8519	1	0.9337	1	1.07	0.53–2.17	1	-0.00547
7	*IL6*	rs1474347	22768124	*C*	*A*	0.1617	1	0.5455	1	1.87	0.78–4.50	1	0.00398
7	*IL6*	rs1548216	22769773	*C*	*G*	0.8231	1	0.9337	1	1.07	0.61–1.86	1	-0.00433
7	*IL6*	rs1818879	22772727	*A*	*G*	0.0091*	0.2542	0.0636	0.2496	2.06	1.20–3.56	0.4504	0.02987
7	*IL6*	rs2066992	22768249	*T*	*G*	0.3577	1	0.8088	1	1.39	0.69–2.78	1	-0.00258
7	*IL6*	rs2069837	22768027	*G*	*A*	0.1754	1	0.5455	1	0.65	0.35–1.21	0.1147	0.00748
7	*IL6*	rs2069855	22772624	*C*	*T*	0.8764	1	0.9337	1	1.07	0.46–2.51	1	-0.00504
12	*IFNG*	rs2069705	68555011	*A*	*G*	0.3858	1	0.8088	1	1.21	0.78–1.88	0.3431	-0.00064
12	*IFNG*	rs2069722	68548953	*A*	*G*	0.4910	1	0.8088	1	0.69	0.24–1.97	1	-0.00276
12	*IFNG*	rs2069728	68547784	*T*	*C*	0.7634	1	0.9337	1	0.93	0.58–1.49	0.3854	-0.00473
12	*IFNG*	rs2430561	68552522	*A*	*T*	0.0729	1	0.3402	1	1.89	0.94–3.80	0.5307	0.01195
12	*IFNG*	rs78554979	68554636	*C*	*T*	0.9004	1	0.9337	1	0.96	0.49–1.89	0.2102	-0.00468
16	*HP*	rs8062041	72088964	*T*	*C*	0.5508	1	0.8569	1	0.88	0.58–1.33	0.6999	-0.00241
22	*APOL1*	rs71785313	36661916	DEL	INSERT	0.0070*	0.1973	0.0636	0.2496	0.39	0.20–0.78	0.3534	0.02888
22	*APOL1*	rs73885319	36661906	*G*	*A*	0.0056*	0.1570	0.0636	0.2496	2.09	1.24–3.53	1	0.03883
22	*MIF*	rs12483859	24234807	*C*	*T*	0.0077*	0.2157	0.0636	0.2496	1.86	1.18–2.95	0.1429	0.03028
22	*MIF*	rs34383331	24238079	*A*	*T*	0.8246	1	0.9337	1	1.06	0.62–1.81	1	-0.00513
22	*MIF*	rs36086171	24235455	*G*	*A*	0.4467	1	0.8088	1	1.24	0.72–2.13	0.3772	-0.00240

CHR: Chromosome number, SNP: single nucleotide polymorphism, BP: Physical position (base-pair in GRCh37), A1: Minor allele name, A2: Major allele name, P: Exact p-value, BONF: Bonferroni corrected p-value, FDR: false discovery rate, OR: Estimated odds ratio (for A1), CI_95_: 95% confidence interval, HWE: Hardy-Weinberg Equilibrium p-value

* P-value significant

DEL: deletion of 6 base pair

INSER: insertion of 6 base pair.

### Association study

The *APOL1* rs73885319 polymorphism is one part of a two SNP haplotype, with derived alleles designated “G1” composed of two tightly linked coding variants rs73885319 (S342G) and rs60910145 (I384M) non-synonymous in the last exon of *APOL1*. The derived allele of rs71785313 called *APOL1* G2 *APOL1* is a 6 base pair deletion, removing amino acids N388 and Y389. Wild type APOL1 is known as G0. APOL1 alleles G1 and G2 are independent [28]. The distribution of *APOL1* G1 and *APOL1* G2 in the present study were significantly different in latent infections compared to both cases and controls (Tables 3 and 4). The *APOL1* G2 allele carriers had a higher risk of developing HAT after infection by *T*. *b*. *gambiense* than the *APOL1* G0 individuals (p = 0.0011, OR = 2.70, CI_95_ = [1.49–4.91], BONF = 0.0301). Subjects carrying the *APOL1* G1 (p = 0.0005, OR = 0.45, CI_95_ = [0.29–0.70], BONF = 0.0129) had an increased risk of developing a latent infection but reduced risk of progressing from latent infection to active HAT than *APOL1* G0 (Table 3).

**Fig 1:**
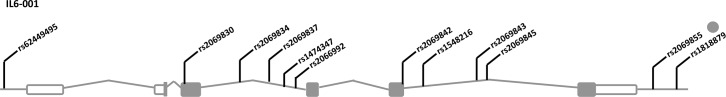
Schematic of single nucleotide polymorphisms of *Interleukin-6* selected from 2,000bp up and downstream (5’ and 3’) of the transcript region.

An association was observed at IL6 rs1818879 (Fig 1), indicating that subjects with latent infections carrying the *A* allele had a lower risk of progressing to active HAT (p = 0.0001, OR = 0.39, CI_95_ = [0.24–0.63], BONF = 0.0034) (Table 3).

The distribution of the *MIF* rs36086171 *G* allele differed between cases and controls (BONF = 0.6697, p = 0.0239, OR = 1.65, CI_95_ = [1.07–2.53]), and *MIF* rs12483859 *C* allele between latent infections and Controls (BONF = 0.2157, p = 0.0077, OR = 1.86, CI_95_ = [1.18–2.95]) but these did not remain significant after Bonferroni correction (Tables 2 and 4).

No statistically significant differences were observed in allele frequency for the polymorphisms of other genes (*IL4*, *IL8*, *HLA-G*, *TNFA*, *HP*, *IFNG* and *MIF*) between cases and controls; cases and latent infection or latent infection and controls in all the analyses.

## Discussion

Association analysis’s undertaken in this study allow us to investigate genetic associations of candidate genes polymorphisms with HAT in a Guinean population.

The main findings of our study are that the A allele of *IL6* rs1818879 and the G allele of *APOL1* G1 appear to be associated with a higher risk of developing a latent infection but a lower risk of progressing from latent infection with undetectable parasitaemia to active disease. These alleles thus seem to provide some degree of protection for individuals with latent infections, providing the ability to maintain infection levels that are undetectable by microscopy. However, the *APOL1* G2 allele increased the risk of progressing from latent infection to active HAT. The associations with the *APOL1* G1 and G2 polymorphisms confirm our previous observations of these SNPs with a more limited sample [20], they were genotyped again in this study as part of the larger multi-country TrypanoGEN consortium study, on an extensive sample from Guinea. Cooper *et al*. found an association between G2 and HAT and Controls in *T*. *b*. *rhodesiense* in Uganda [20].

APOL1 is a component of the trypanosome lytic factor (TLF) of human serum that confers resistance to *T*. *b*. *brucei* [29,30]. APOL1forms pores in the parasite endolysosomal membranes and triggers lysosome swelling which leads to trypanolysis [31]. *APOL1* expression is also induced by *T*. *b*. *gambiense* infection enhancing its lytic activity [32]. African trypanosomes, except *T*. *b*. *gambiense* and *T*. *b*. *rhodesiense* are lysed by APOL1. These two subspecies can resist lysis by APOL1 because they express the serum resistance glycoprotein (TgsGP) and serum resistance-associated protein (SRA), respectively [33–35]. *T*. *b*. *rhodesiense* SRA inhibits APOL1 by direct binding but TgsGP acts by limiting uptake of APOL1. *T*. *b*. *gambiense* (group 1) also can resist TLF-1 killing because coding sequence mutations to the *TbgHpHbR*, reduce expression of Hp/Hb receptor and limit TLF-1 uptake [36]. The mode of action of G1 is unknown but the G2 mutation limits binding of SRA to APOL1 and should therefore make APOL1 G2 lytic to *T*. *b*. *rhodesiense* but this mechanism could not effect *T*. *b*. *gambiense*, which does not have the SRA gene [28,37]. In this study, we found that the 6 base pair deletion in *APOL1* G2 is risk factor for developing an active *T*. *b*. *gambiense* infection from a latent infection.

*IL6* rs1818879 *A* allele carriers had a lower risk of developing the disease. rs1818879 appears to fall within a CCCTC-Binding factor (CTCF) binding site and GTEx reports rs1818879 as an eQTL for AC073072, a novel antisense RNA gene within *IL6* on the opposite strand about which little is known [38]. CTCF is a zinc finger protein that can be involved in activation or repression of gene expression and the disruption of this binding site may account for the eQTL associated with AC073072 [39]. Although the mechanism remains unclear, these data suggest that rs1818879 may be a functional polymorphism and not just a marker for differences in response to infection.

It has been shown that IL6 could play a role on the modification of blood brain barrier permeability *in vitro* together with other pro-inflammatory cytokines such as IL1 and TNFA in blood and/or in CNS [40]. IL6 plasma levels were found to be significantly higher in individuals with latent infection from Guinea as compared to controls or HAT patients [17]. Girard *and al*. (2005) showed that IL6 synthesis was induced in bone marrow by *T*. *b*. *gambiense in vitro* [41]. Therefore, Il6 appears as an important inflammatory cytokine mediating *T*. *b*. *gambiense* response and suggest that IL6 could play a role in the phenomenon of latent infections without parasitological confirmation. The result obtained with *IL6* rs1818879 in our study is consistent with the data from a candidate gene association study in DRC, where rs2069849 in *IL6* was shown to be associated with a decreased risk of developing the disease [16].

Our data show that the frequency of the *G* minor allele of *MIF* rs36086171 was higher in cases than in controls (uncorrected p = 0.0239, OR = 1.65, CI_95_ = [1.07–2.53]) and *MIF* rs12483859 *C* allele in latent infections than in controls (uncorrected p = 0.0077, OR = 1.86, CI_95_ = [1.18–2.95]). MIF is an important component of the host response implicated in the antimicrobial response and promotes the secretion and activation of pro-inflammatory cytokines, by immune cells [42,43]. Low expression of *MIF* has been described as favoring infection and disease progression in leishmaniasis [44]. We did not find a significant difference after correction (BONF = 0.0588), but it is known that this gene can contribute to disease development in a mice experimental model [45].

In conclusion, this study provides further evidence that the clinical diversity of sleeping sickness is partly due to the genetic diversity of the hosts. Our data demonstrate that the outcome of the disease is affected by three polymorphisms (*APOL1* G1, G2 and *IL6* rs1818879) in the Guinean population. This study was performed in the framework of the TrypanoGEN consortium to systematically investigate the role of host genetics in disease susceptibility and progression across East and West African populations. Further studies need to be conducted to confirm these results and to determine the mechanisms by which these alleles affect disease progression and outcome in HAT and could lead to the discovery of human natural resistance mechanisms and thus to the development of new tools for the control of this neglected tropical disease.
